# Effects of faecal microbiota transplantation on the small intestinal mucosa in systemic sclerosis

**DOI:** 10.1093/rheumatology/kead014

**Published:** 2023-01-23

**Authors:** Noemi Strahm, Henriette Didriksen, Håvard Fretheim, Øyvind Molberg, Øyvind Midtvedt, Inger Nina Farstad, Tore Midtvedt, Knut E A Lundin, Lars Aabakken, Przemysław Błyszczuk, Oliver Distler, Gabriela Kania, Anna-Maria Hoffmann-Vold

**Affiliations:** Center of Experimental Rheumatology, Department of Rheumatology, University Hospital Zurich, University of Zurich, Schlieren, Switzerland; Department of Rheumatology, Oslo University Hospital, Rikshospitalet, Oslo, Norway; Institute of Clinical Medicine, University of Oslo, Oslo, Norway; Department of Rheumatology, Oslo University Hospital, Rikshospitalet, Oslo, Norway; Department of Rheumatology, Oslo University Hospital, Rikshospitalet, Oslo, Norway; Institute of Clinical Medicine, University of Oslo, Oslo, Norway; Department of Rheumatology, Oslo University Hospital, Rikshospitalet, Oslo, Norway; Institute of Clinical Medicine, University of Oslo, Oslo, Norway; Department of Pathology, Oslo University Hospital, Rikshospitalet, Oslo, Norway; Department of Microbiology, Tumor and Cell Biology, Karolinska Institutet, Stockholm, Sweden; Institute of Clinical Medicine, University of Oslo, Oslo, Norway; Department of Gastroenterology, Oslo University Hospital, Rikshospitalet, Oslo, Norway; Institute of Clinical Medicine, University of Oslo, Oslo, Norway; Department of Gastroenterology, Oslo University Hospital, Rikshospitalet, Oslo, Norway; Center of Experimental Rheumatology, Department of Rheumatology, University Hospital Zurich, University of Zurich, Schlieren, Switzerland; Department of Clinical Immunology, Jagiellonian University Medical College, Cracow, Poland; Center of Experimental Rheumatology, Department of Rheumatology, University Hospital Zurich, University of Zurich, Schlieren, Switzerland; Center of Experimental Rheumatology, Department of Rheumatology, University Hospital Zurich, University of Zurich, Schlieren, Switzerland; Department of Rheumatology, Oslo University Hospital, Rikshospitalet, Oslo, Norway; Institute of Clinical Medicine, University of Oslo, Oslo, Norway

**Keywords:** SSc, gastrointestinal tract, faecal microbiota transplantations, immunohistochemistry, RNA-sequencing

## Abstract

**Objectives:**

In SSc, gastrointestinal tract (GIT) involvement is a major concern, with no disease-modifying and limited symptomatic therapies available. Faecal microbiota transplantation (FMT) represents a new therapeutic option for GIT-affliction in SSc, showing clinical promise in a recent controlled pilot trial. Here, we aim to investigate effects of FMT on duodenal biopsies collected from SSc patients by immunohistochemistry and transcriptome profiling.

**Methods:**

We analysed duodenal biopsies obtained pre-intervention (week 0) and post-intervention (weeks 2 and 16) from nine SSc patients receiving an intestinal infusion of FMT (*n* = 5) or placebo (*n* = 4). The analysis included immunohistochemistry (IHC) with a selected immune function and fibrosis markers, and whole biopsy transcriptome profiling.

**Results:**

In patients receiving FMT, the number of podoplanin- and CD64-expressing cells in the mucosa were lower at week 2 compared with baseline. This decline in podoplanin- (*r* = 0.94) and CD64-positive (*r* = 0.89) cells correlated with improved patient-reported lower GIT symptoms. Whole biopsy transcriptome profiling from week 2 showed significant enrichment of pathways critical for cellular and endoplasmic reticulum stress responses, microvillus and secretory vesicles, vascular and sodium-dependent transport, and circadian rhythm. At week 16, we found enrichment of pathways mandatory for binding activity of immunoglobulin receptors, T cell receptor complexes, and chemokine receptors, as well as response to zinc-ions. We found that 25 genes, including Matrix metalloproteinase-1 were upregulated at both week 2 and week 16.

**Conclusion:**

Combining selective IHC and unbiased gene expression analyses, this exploratory study highlights the potential for disease-relevant organ effects of FMT in SSc patients with GIT involvement.

**Trial registration:**

ClinicalTrials.gov, http://clinicaltrials.gov, NCT03444220.

Rheumatology key messages

- Faecal microbiota transplantations (FMT) treatment with Anaerobic Cultivated Human Intestinal Microbiome (ACHIM) might have an effect on duodenal inflammation, fibrosis, and lymphangiogenesis.
- There might be a correlation with improved patient-reported lower GIT symptoms.
- Changed expressions of several genes were observed in duodenal samples of SSc patients receiving FMT.

## Introduction

SSc is a multi-organ disease with frequent gastrointestinal tract (GIT) involvement representing a major clinical concern [1–3]. All parts of the GIT are susceptible to disease-related pathologies. The mechanisms by which SSc affects the various parts of the GIT are not well understood, explaining why there are limited specific therapies available for GIT involvement [4–7].

Major advances in gut microbiome sequencing have provided evidence for alteration in the bacterial ecosystem across a number of human diseases, including SSc and primary GIT diseases [8]. Several studies across a number of geographic locations have shown altered gut microbiota in SSc patients with and without GIT symptoms [5, 9–12]. Based on the hypothesis that altered gut microbiota triggers and drives pathology, and is associated with GIT symptoms, faecal microbiota transplantation (FMT) has been suggested as a therapeutic alternative, with early trials showing promising results [13, 14]. We explored the clinical effects of FMT in SSc patients with GIT involvement in a pilot trial, in which nine SSc patients received Anaerobic Cultivated Human Intestinal Microbiome (ACHIM) or placebo instillation. We found a possible improvement in lower GIT symptoms [15]. The only other FMT trial conducted in rheumatic diseases, for active peripheral PsA, seemed to be safe, and FMT did not appear to be inferior compared with placebo [16]. The mechanisms by which FMT exerts its effects in rheumatic diseases are not known. Indications come from other human GIT diseases, including irritable bowel syndrome (IBS), for which improvement in GIT symptoms after FMT was correlated with changes in duodenal enteroendocrine cell density [17].

In patients with IBD, FMT acted by influencing the abundance of specific members of the gut microbial community and by changes in host immunological pathways, which makes histological analysis of inflammatory, fibrotic, and lymphangiogenic markers in duodenal samples from SSc patients receiving FMT tempting [18].

In this study, we assessed duodenal biopsies collected from SSc patients pre- and post-treatment with FMT or placebo, and from SSc patients with archived duodenal tissue samples. Additionally, we investigated the effects of FMT on duodenal transcriptome profiling and cellular composition in these patients.

## Methods

### Study population and biosamples

In the ReSScue pilot trial, we randomized nine female patients with lcSSc and lower and upper GIT symptoms at Oslo University Hospital (OUH) to either ACHIM (*n* = 5) or placebo (*n* = 4) instillation by gastro duodenoscopy with biosampling [15]. The number of participants was restricted to the pilot nature of study. Patients had no dietary requirements but were briefed not to change their diet. One patient from each group was on a gluten-free diet, and one from the ACHIM group was on a low-FODMAP diet, while the rest had a normal Norwegian diet. The intake of probiotics was stopped 4 weeks prior to the first intervention. The use of antibiotics during the study period or 3 months prior was an exclusion criterion, and none of the patients had IVIG administration [15]. Detailed inclusion and exclusion criteria can be found in the recently published pilot trail [15].

Biosampling and assessment of patient-reported GIT symptoms by the University of California, Los Angeles Scleroderma Clinical Trial Consortium GIT 2.0 (UCLA GIT) score were collected pre- (week 0) and post-intervention (weeks 2 and 16) [15, 19–21]. All duodenal biopsies were taken before noon on the day of FMT. Thereafter, the biopsies were processed according to routine standards before paraffin embedding, sectioning into 3-µm tissue sections, and staining with Haematoxylin and Eosin.

In addition, archived duodenal biopsies from 17 SSc patients followed at the Department of Rheumatology OUH who had undergone a gastroscopy with biosampling on clinical indication were analysed in order to characterize baseline differences in duodenal histology of SSc patients and the ReSScue pilot cohort. The biopsies were collected 5–15 years prior to this study, and the clinical indications for gastroscopy were investigation of celiac disease, reflux disease or eosinophilic esophagitis. All patients fulfilled the 2013 ACR/EULAR SSc classification criteria [22].

The Regional Committee for Medical and Health Research Ethics (REK) in Oslo approved the study (No: 2016/1529) and the use of the Norwegian CTD and vascular registry (NOSVAR) research biobank (No: 2016/119). The pilot trial followed the Declaration of Helsinki and is registered at clinicaltrials.gov (NCT03444220). Prior to the study start, all participants gave informed written consent. The Department of Pathology at OUH approved the sharing of biological material (No: 2020–31).

### Immunohistochemistry

To investigate the effect of FMT by ACHIM on inflammatory, fibrotic, and lymphangiogenic factors in the duodenum, we performed immunohistochemistry (IHC) on duodenal biopsies from the ReSScue trial patients and the 17 SSc controls. The biopsies were stained with CD38, CD3, CD8, CD64, podoplanin, VEGFR3, αSMA and Sirius Red. Detailed information on staining protocols, antibodies, and data analysis is explained in the Supplementary Methods and Supplementary Table S1, available at *Rheumatology* online.

### Deep RNA sequencing

RNA sequencing was performed on total RNA extracted from duodenum biopsies isolated from ReSScue patients pre- and post-intervention. The detailed protocol for gene expression analysis is explained in the Supplementary Methods, available at *Rheumatology* online.

### Statistical analysis and bioinformatics

Analyses were conducted using IBM SPSS 26, STATA 17 and GraphPad Prism 8. The Pearson χ^2^ test, the Fisher exact test, and the independent sample *t* test were used as appropriate. For correlation analysis, we applied Pearson correlation (*r*), and calculated the correlation between mean staining/area and mean lower UCLA GIT score in the ReSScue pilot patients receiving ACHIM or placebo. Next, we assessed correlations of the relative abundance of faecal bacteria by 16S rRNA sequencing found altered in the pilot FMT study (genus *Bacterioides*, genus *Dialister*, family Lachnospiraceae, genus *Agathobacter* and genus *Phascolarctobacterium*) [15] and reported to be associated with GIT symptoms in various diseases with mean staining/area and gene expression (Supplementary Methods and Table S2, available at *Rheumatology* online). This is a proof-of-concept study with a low patient number, and therefore no power calculation and analysis for normality has been conducted.

## Results

While analyses of pre- and post-intervention faecal samples from the ReSScue pilot trial indicate that FMT by ACHIM caused a change in GIT bacterial composition persisting for at least 16 weeks [15], the potential effects of FMT on the small intestinal mucosa remain unknown. To address this issue, we assessed duodenal biopsies obtained from the trial patients at weeks 0, 2 and 16. Analyses on duodenal samples included IHC, focusing on cellular markers relevant for various aspects of the SSc pathogenesis, including inflammatory, fibrotic and lymphangiogenic processes, and transcriptomic profiling. For comparison, we included IHC analyses of archived duodenal samples from 17 SSc controls. Characteristics and demographics of the pilot trial patients and control patients are shown in Table 1.

**Table 1.** Characteristic and demographic data for all SSc patients from the ReSScue FMT pilot trial, receiving FMT or placebo, and from unselected SSc control patients

	ReSScue patients (*n* = 9)	FMT intervention (*n* = 5)	Placebo intervention (*n* = 4)	SSc control patients (*n* = 17)
Age at sampling, mean years (s.d.)	62 (5.7)	58 (5.6)	66 (1.5)	52 (4.8)
Female, *n* (%)	9 (100)	5 (100)	4 (100)	17 (100)
Disease duration, mean years (s.d.)	12.0 (10.6)	7.4 (6.7)	17.8 (12.6)	5.3 (4.2)
lcSSc, *n* (%)	9 (100)	5 (100)	4 (100)	10 (59)
ACA, *n* (%)	8 (89)	5 (100)	3 (75)	8 (47)
Anti-Scl-70 antibody, *n* (%)	0 (0)	0 (0)	0 (0)	2 (15)
GIT involvement				
Oesophagal involvement, *n* (%)	9 (100)	5 (100)	4 (100)	13 (76)
Dysphagia, *n* (%)	8 (89)	4 (80)	4 (100)	8 (47)
Reflux disease, *n* (%)	4 (44)	3 (60)	1 (25)	11 (73)
GAVE, *n* (%)	0 (0)	0 (0)	0 (0)	2 (12)
Diarrhoea, *n* (%)	5 (56)	3 (60)	2 (50)	5 (29)
Distension/Bloating, *n* (%)	7 (78)	5 (100)	2 (50)	10 (59)
Obstipation, *n* (%)	7 (78)	4 (80)	3 (75)	5 (29)
Soilage/Incontinence, *n* (%)	1 (11)	0 (0)	1 (25)	4 (24)
UCLA-GIT Score	0.7 (0.5)	0.7 (0.4)	0.8 (0.6)	0.7 (0.8)
Lung involvement				
ILD, *n* (%)	2 (22)	2 (40)	0 (0)	6 (35)
FVC, mean (s.d.), predicted	86.8 (28.2)	78.2 (34.8)	97 (10.4)	97.6 (27)
FEV1, mean (s.d.)	83.9 (14.3)	86.6 (13.5)	80.5 (14.5)	81.7 (18.5)
DLCO, mean (s.d.)	74.4 (13.4)	76.4 (12)	72 (18)	63.9 (22.1)
PAH, *n* (%)	0 (0)	0 (0)	0 (0)	3 (18)
Cutaneus manifestation				
Digital ulcers, *n* (%)	0 (0)	0 (0)	0 (0)	8 (47)
Calcinosis, *n* (%)	3 (33)	2 (40)	1 (25)	7 (41)
Telangiectasia, *n* (%)	6 (67)	3 (60)	3 (75)	13 (76)
mRSS, mean (s.d.)	4.9 (4.8)	4.3 (3.8)	5.5 (5.6)	7 (5.5)
Renal involvement				
Scleroderma Renal Crisis, *n* (%)	0 (0)	0 (0)	0 (0)	1 (6)
Medication				
Proton Pump Inhibitor, *n* (%)	7 (77)	3 (60)	4 (100)	2 (12)
MMF, *n* (%)	1 (11)	1 (20)	0 (0)	3 (17)
MTX, *n* (%)	1 (11)	0 (0)	1 (25)	2 (12)
CSs, *n* (%)	2 (22)	1 (20)	1 (25)	4 (24)
Rituximab, *n* (%)	1 (11)	0 (0)	1 (25)	1 (6)

mRSS: modified Rodnan Skin Score; GIT: gastrointestinal tract; GERD: gastroesophageal reflux disease; GAVE: gastric antral vascular ectasia; UCLA-GIT score: University of California Los Angeles Gastrointestinal score; ILD: interstitial lung disease; FMT: faecal microbiota transplantation; FVC: forced vital capacity; FEV1: forced expiratory volume in 1 s; DLCO: diffuse capacity of the lung carbon monoxide; PAH: pulmonary arterial hypertension.

### FMT treatment effects on duodenal markers

**Figure 1.**
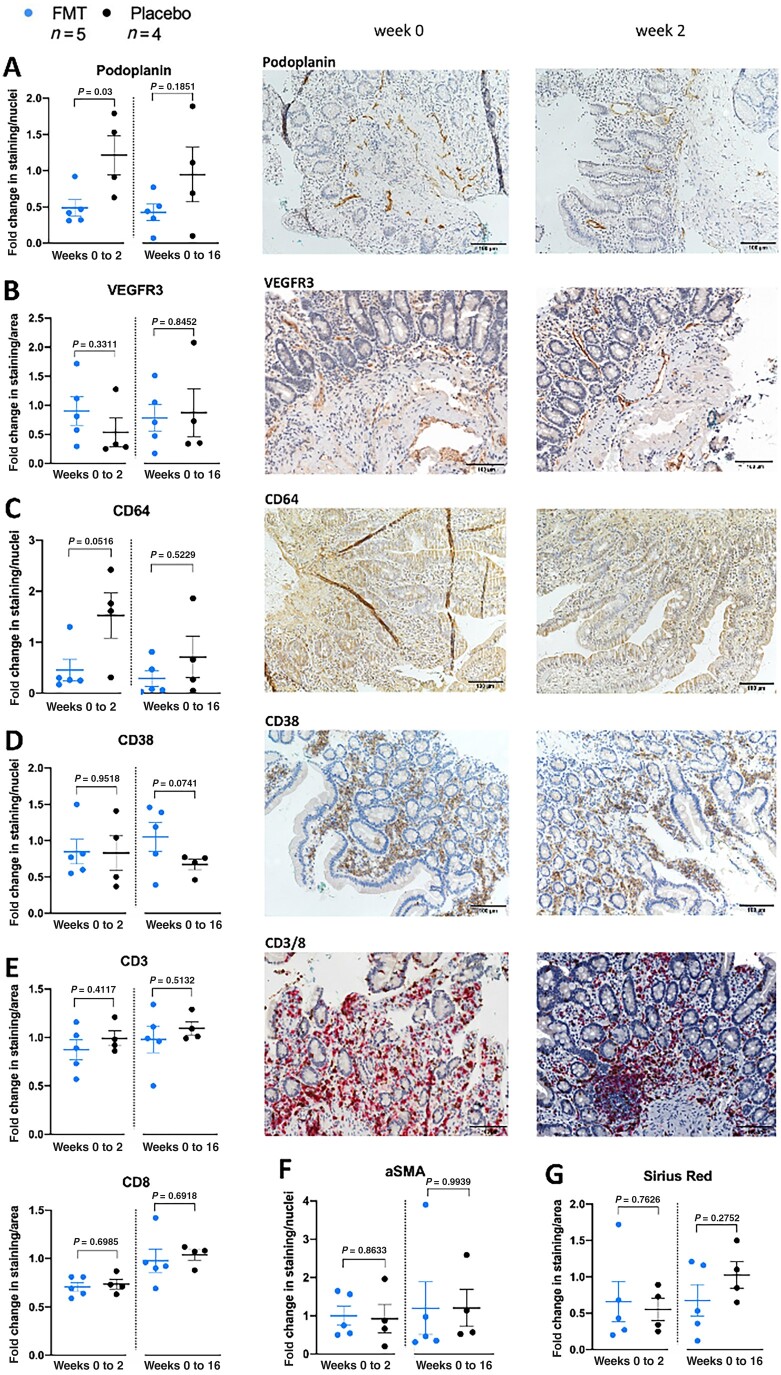
Immunohistochemical analysis and representative pictures of duodenal samples of ReSScue patients (FMT *n* = 5, Placebo *n* = 4). Duodenal biopsies were stained with antibodies for podoplanin, VEGFR3, CD64, CD38, CD3, CD8, and αSMA, and Sirius Red. (**A–G**) Staining/area or nuclei depicted as fold change between weeks 0 and 2 (fold change 1) and between weeks 0 and 16, respectively, for patients who received FMT (left) and placebo (right) (unpaired *t* test). (**A–E**) Representative pictures at weeks 0 (left) and 2 (right) in magnification ×20. All pictures are from patients who received FMT. FMT: faecal microbiota transplantation

We first wanted to assess the effect of FMT on the duodenum of SSc patients, as dysbiosis may play a crucial role in the development of SSc GIT symptoms as shown in IBD and IBS [17, 18]. Histological analysis of the duodenal samples showed a good representation of villi and crypts, while the mucosa and submucosa were fully represented in most of the patients (Supplementary Fig. S1 and Supplementary Table S3, available at *Rheumatology* online). As shown in Fig.1A –G and Supplementary Fig. S2 (available at *Rheumatology* online), we observed a reduction of podoplanin (gp38) (*P* =* 0*.*03*) and CD64 (*P = 0.052*) expression at week 2 and a tendency of sustained lower podoplanin expression at week 16 (*P = 0.19*) in FMT patients. We saw that podoplanin was expressed in the lymphatic vessels and CD64 was mostly expressed in the epithelium. The ratios of other markers, such as CD3 and CD8 (expressed on T cells), VEGFR3 (expressed in lymphatic vessels), αSMA (expressed in smooth muscle cells), Sirius Red (stained collagen fibres), and CD38 (mostly expressed on plasma cells in the epithelium and on lymphocytes), remained unchanged between the FMT-treated and placebo arms at week 2.

### Correlation of histological changes in the duodenum with clinical parameters and microbiota composition

In our previous work, we showed that FMT by ACHIM may be associated with an improvement in lower GIT symptoms, including distension/bloating, diarrhoea, and faecal soilage, reported by the UCLA GIT score [15]. To investigate whether the change in patient-reported symptoms correlated with changes in IHC results, we performed correlation analysis between mean fold change of staining ratios for all markers, and a modified version of the UCLA GIT score, including only lower GIT symptoms.

In all stainings, a change from weeks 0 and 2 was observed in FMT patients, while in the placebo patients, a tendency towards clustering of measurements was observed (Supplementary Fig. S3, available at *Rheumatology* online). In the patients receiving FMT, the correlation between lower UCLA GIT score and staining was strong for both podoplanin (FMT: *r* = 0.94, Placebo: *r* = 0.18) and CD64 (FMT: *r* = 0.89, Placebo: *r *= 0.35). Importantly, we showed a significant reduction in podoplanin and CD64 expression in these patients (Supplementary Fig. S3, available at *Rheumatology* online).

When correlating staining/area of podoplanin and CD64 with relative abundance of bacteria, we only found a significantly strong correlation in the FMT patients for the genus *Phascolarctobacterium* with podoplanin (*r* = 0.99, *P* =* *0.02) and CD64 (*r* = 1.00, *P* < 0.01) (Supplementary Fig. S4, available at *Rheumatology* online).

**Figure 2.**
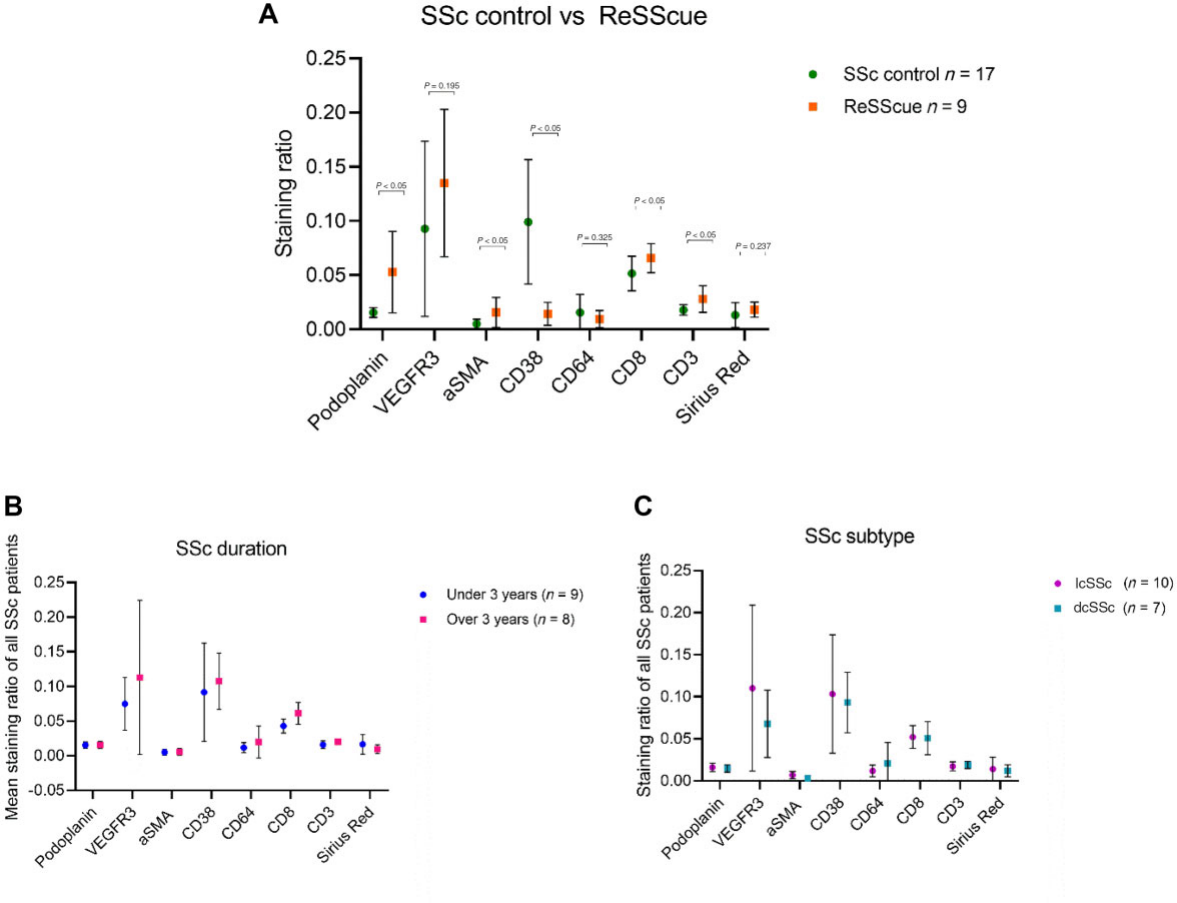
Immunohistochemical analysis and clinical correlation of unselected SSc patients (*n* = 17) and the ReSScue patients (*n* = 9). (**A**) Mean ratios of unselected SSc patients (circle) and ReSScue patients (square). ReSScue patient data are depicted from week 0; therefore, FMT and placebo patients are not marked separately. The values of CD3 and CD8 were divided by 10, as the ratios were higher due to higher staining intensity. The bar indicates standard deviation, and *P* was calculated with an unpaired *t* test. (**B**) Mean staining ratios and standard deviations for individual markers of SSc control patients with disease duration ≤3 years (circle) and >3 years (square). (C) Mean staining ratios and standard deviations for individual markers of SSc control patients having lcSSc (circle) and dcSSc (square) disease. FMT: faecal microbiota transplantation

To assess whether patients in the ReSScue trial were representative of SSc, archived samples from 17 SSc controls who had undergone a gastroduodenoscopy were analysed. Table 1 shows small differences between the ReSScue pilot patients and the SSc controls. The total GIT score of the SSc controls and the ReSScue trial patients was comparable [0.7 (0.8) *vs* 0.7 (0.5)]. However, lower GIT involvement was more frequent in the ReSScue trial patients compared with the SSc controls. Histological analysis showed a good representation of villi, crypts, mucosa, and submucosa. As shown in Fig. 2, the IHC analysis of the ReSScue patients and SSc controls revealed heterogeneous results for all stainings, with the largest variations in VEGFR3 and CD38. IHC results differed significantly between the groups (*P* < 0.05) for podoplanin, αSMA, CD38, CD8 and CD3, with higher staining ratios in ReSScue patients in all, except CD38 (Fig. 2A). Although GIT disease often evolves over the disease course, the correlation of staining ratios of SSc controls with disease duration ≤3 years (short) and >3 years (longer) showed no correlation (Fig. 2B). No correlation of IHC with disease subtype (lcSSc, dcSSc) was observed (Fig. 2C).

### FMT treatment effects on duodenal transcriptome profiling

RNA sequencing was performed on total RNA extracted from duodenum biopsies isolated from placebo- and FMT-treated SSc patients at weeks 0, 2 and 16. The range of reads/sample was >23 million to >35 million.

**Figure 3.**
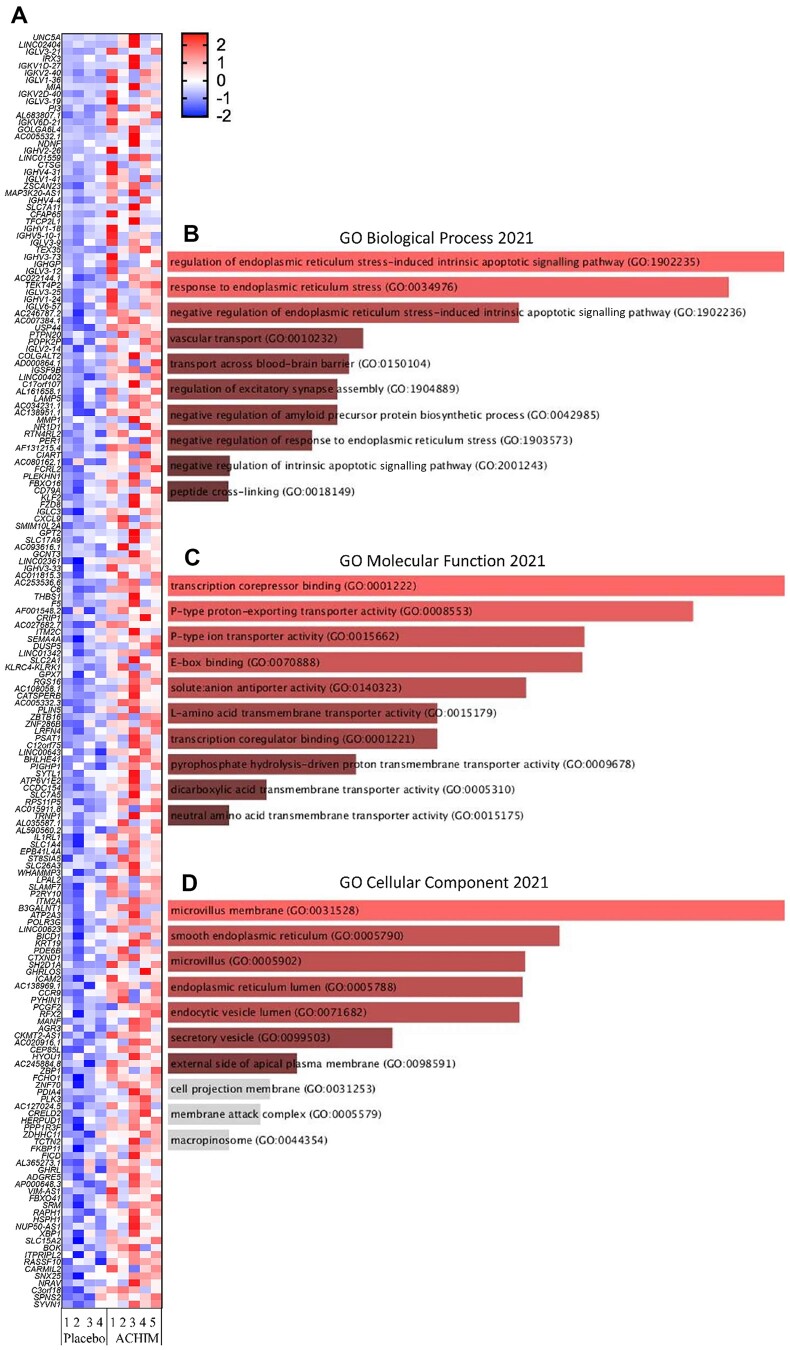
Transcriptomic analysis of duodenal samples from SSc patients 2 weeks after treatment with FMT or placebo, Part 1. Bulk RNA sequencing was performed on duodenal biopsies from SSc placebo (4) *vs* FMT (5) patients. (**A**) The heat map and (**B–D**) gene ontology (GO) analysis of significantly upregulated genes in duodenal biopsies from patients 2 weeks after treatment with FMT or placebo. The EnrichR software performed pathway enrichment analysis of differentially expressed genes (*P* ≤ 0.05, log2 ratio ≥ 0.5). FMT: faecal microbiota transplantation

Specifically, we detected 328 differentially expressed genes (179 upregulated, 149 downregulated) in the FMT group compared with the placebo group at week 2 (Fig. 3A; Supplementary Fig. S5, available at *Rheumatology* online) and 467 differentially expressed genes (297 upregulated, 170 downregulated) in the FMT group compared with the placebo group at week 16 (Supplementary Figs S6 and S7, available at *Rheumatology* online) (*P* ≤ 0.05, log2 ≥ 0.5). Within the upregulated genes in the FMT group at week 2, we observed a significant enrichment of pathways critical for the endoplasmic reticulum (ER) stress, vascular transport, microvillus, and secretory vesicles (Fig. 3B–D). Among genes implicated in those processes, we observed higher expression of the transcription factors (*KLF2* and *XBP1*) important for the proper functioning of the immune system and cellular stress response, and genes involved in the sodium-dependent glucose and galactose transport (*SLCs)*. Integral membrane protein 2A (*ITM2A*), involved in the activation of T cells in the immune system, and the enzyme-intestinal alkaline phosphatase (*ALPI*) gene, a component of the gut mucosal defence system, which may function in the detoxification of lipopolysaccharide and the prevention of bacterial translocation in the gut, were also higher expressed (Fig. 3A).

In the FMT group compared with the placebo group at week 2, we observed downregulation of *HLA* genes, which are upregulated in gastrointestinal and rheumatic disorders (Supplementary Fig. S5, available at *Rheumatology* online).

Within genes upregulated in the FMT group at week 16, we observed a significant enrichment of pathways critical for the response to zinc-ion, the Ig receptor, chemokine receptor binding and activity, and the T cell receptor complex (Supplementary Fig. S6, available at *Rheumatology* online). Within downregulated genes in the FMT group at week 16, we found enrichment of pathways crucial for extracellular structural organization, collagen-containing extracellular matrix, the adrenergic and glutamate receptor signalling pathways, and VEGF receptor binding (Supplementary Fig. S7, available at *Rheumatology* online).

**Figure 4.**
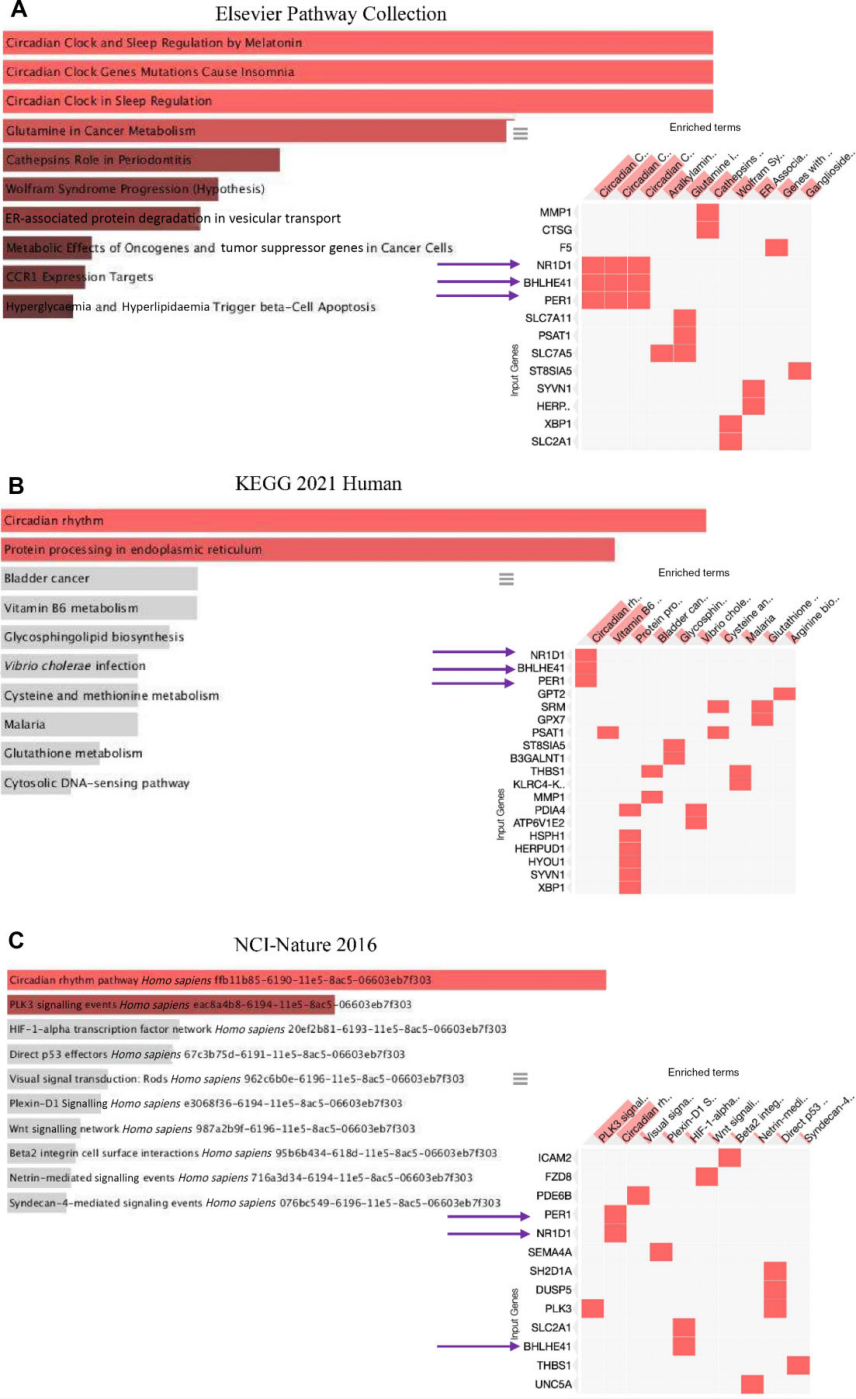
Transcriptomic analysis of duodenal samples from SSc patients 2 weeks after treatment with FMT or placebo, Part 2. Pathway analysis of significantly upregulated genes in duodenal biopsies from SSc patients after treatment with FMT or placebo at week 2 (*P* ≤ 0.05, log2 ratio ≥ 0.05). The analysis was performed with the comprehensive gene set enrichment web server EnrichR for Pathways [database: (**A**) Elsevier Pathway Collection, (**B**) KEGG2021 Human, (**C**) NCI-Nature 2016] of significantly upregulated genes in samples from SSc patients treated with FMT or placebo at week 2. Arrows indicate upregulated expression of *NR1D1*, *PER1* and *BHLME41*. FMT: faecal microbiota transplantation

In the FMT group at week 2, we found a significant enrichment of the pathways for the circadian clock and rhythm within the upregulated genes. Among the genes involved in these pathways, we detected higher expression of *NR1D1*, *PER1* and *BHLME41* genes (Fig. 4A–C).

**Figure 5.**
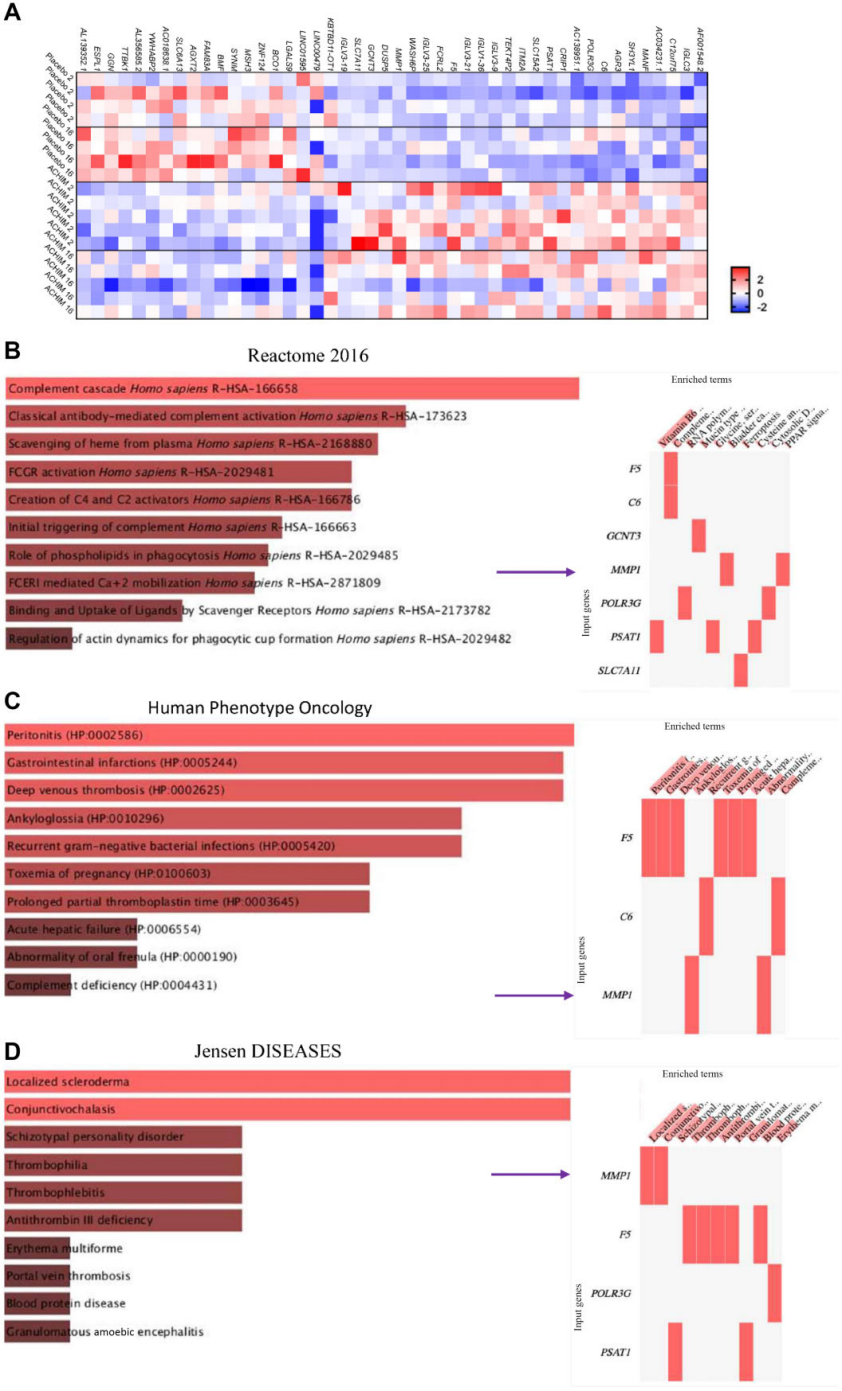
Transcriptomic comparative analysis of duodenal samples from SSc patients treated with FMT or placebo between weeks 2 and 16. Bulk RNA sequencing was performed on duodenal biopsies from SSc placebo (*n* = 4) *vs* FMT (*n* = 5) patients. (**A**) The heat map and (**B–D**) pathway analysis of significantly upregulated and downregulated genes. The analysis was performed with the comprehensive gene set enrichment web server EnrichR for Pathways [database: (**A**) Reactome 2016, (**B**) Human Phenotype Oncology, (**C**) Jensen DISEASES] of significantly upregulated genes in samples from SSc patients treated with FMT at weeks 2 and 16 (*P* ≤ 0.05, log2 ratio ≥ 0.5). Arrows indicate upregulated expression of *MMP1*. FMT: faecal microbiota transplantation

Essentially, there are 40 commonly deregulated genes in the FMT group at weeks 2 and 16 (25 upregulated, 15 downregulated) (Fig. 5; Supplementary Fig. S8, available at *Rheumatology* online). Within the upregulated genes, we found *MMP-1*, which is an interstitial collagenase involved in the cleavage collagens, types I, II and III.

Correlation with gut microbiome composition showed a tendency to positive correlations between all bacteria families and the top upregulated genes in the FMT group at week 2, including *KLF2*, *SLCA5*, *ITM2*, *PER1*, *BHLHE41* and *MMP1* (Supplementary Fig. S9, available at *Rheumatology* online).

## Discussion

GIT involvement is present in 90% of SSc patients and is a leading cause of morbidity. However, the mechanisms by which SSc affects the various parts of the GIT are poorly understood, and disease-modifying treatment is lacking.

Previously we have shown that FMT-treatment with ACHIM possibly reduces lower GIT symptoms in SSc patients [15], and in PsA FMT did appear to be efficacious, but the mechanisms by which FMT exerts its effects in rheumatic diseases are also not known [16]. In patients with IBS, improvements in GIT symptoms were associated with changes in duodenal enteroendocrine cell density after FMT [17]. These findings motivated us to investigate the effects of FMT in SSc on duodenal transcriptome profiling and cellular composition.

First, we analysed the effect of FMT treatment on duodenal histology but found no obvious morphological changes. With IHC analysis, we saw a significant reduction in podoplanin expression in lymphatic vessels and CD64 expression in the epithelium from weeks 0–2 in the duodenum of FMT-treated patients. CD64 is a high-affinity receptor for IgG (Fcγ-R1) and is constitutively expressed in the intestinal epithelium [23, 24]. Furthermore, it has been implicated that in the context of intestinal mucosal inflammation, CD64 modulates the response against microbial challenges [25]. We hypothesize, that certain gut bacteria in ACHIM may express proteins on the outer membrane, signalling through IgG Fcγ-receptors and reducing inflammation, as shown by reduced CD64 expression on the enterocytes. The expression of the other markers investigated showed minimal changes. The changes were not significant, possibly due to the small sample size, and confirmation in a larger patient cohort is required. The orientation of the tissue on the slides may also affect the staining ratios, especially the staining of collagen fibres by SIR when larger parts of submucosa were represented in the biopsies.

Second, we correlated IHC findings with GIT symptoms at weeks 0, 2 and 16. The staining ratios for podoplanin and CD64 had a strong correlation with lower GIT symptoms in the FMT-treated patients. In the ReSScue pilot, we saw that GIT symptoms initially improved after FMT but tended to increase again after 12 weeks [15]. The initial improvement in GIT symptoms in the present study was accompanied by decreased podoplanin and CD64 expression after two infusions of FMT at week 2, but not at week 16. Interestingly, at week 16, we observed a tendency to express CD38 more strongly on plasma cells, enterocytes, and lymphocytes, which has been linked to intestinal inflammation [26]. This may indicate that repeated FMT treatment should be considered in the future to control GIT symptoms and intestinal inflammation. Apart from the genus *Phascolarctobactererium*, none of the changes in bacterial abundance were significantly correlated with podoplanin, or CD64 expression.

Furthermore, a comparison with 17 unselected SSc patients showed astonishingly heterogeneous results, whereas ReSScue patients revealed higher pro-inflammatory, pro-fibrotic, and lymphangiogenetic markers. We showed that the staining level of podoplanin, αSMA and CD3 is close to zero for the SSc controls. These samples were archived for 5–15 years, and it is unclear how stable these markers are in paraffin blocks over a longer period. This could also indicate that the ReSScue pilot patients had more severe GIT affection than the SSc controls. However, the CD38 expression was higher in the SSc controls, which we cannot clearly explain. The patient characteristic comparison showed that the ReSScue pilot patients reported more frequently lower GIT symptoms, such as diarrhoea. A sub-analysis of SSc controls with lower GIT symptoms did not show specific patterns.

In addition, RNA-sequencing was performed on the duodenal biopsies collected at weeks 0, 2 and 16 from all ReSScue pilot patients. Transcriptomic profiling displayed the gene upregulation of pathways critical for proper functioning of the immune system, cellular stress response, and sodium-dependent glucose and galactose transport, with higher *KLF2*, *XBP1* and *SLC* gene expression in patients receiving FMT compared with placebo at week 2. *KLF2* mediates anti-inflammatory and anti-fibrotic effects in pulmonary fibrosis [27]. Our results may indicate that FMT induces upregulation of *KLF2* and further reduces inflammation and fibrosis in the duodenum, partly supported by the IHC findings in this study. The transcription factor *XBP1* is an essential element of the ER stress response. ER stress mediates development and progression of autoimmune diseases and is related to immune diseases such as RA, atherosclerosis and IBD [28–30]. Deletion of *XBP1* in intestinal epithelial cells causes spontaneous enteritis [31]. Therefore, downregulation of *XBP1* expression in epithelial cells may initiate intestinal inflammation by the cell-specific aberrant ER stress response. In patients receiving FMT, we observed a higher *XBP1* expression compared with those receiving placebo at week 2, which may suggest the involvement of *XBP1* in the anti-inflammatory response. This stress response observed after FMT could be a sign of a direct response in the duodenum from the treatment itself.

*SLC* genes are transporters important for the uptake of biologically active compounds in the intestine, and downregulation of these genes can cause activation of pro-inflammatory signalling [32]. Upregulated expression of *SLC* genes in patients receiving FMT compared with placebo at week 2 may indicate the activation of anti-inflammatory processes.

The transcriptomic data demonstrate significant enrichment of pathways for the circadian clock and rhythm within upregulated genes in the FMT group at week 2. Circadian rhythms regulate cell proliferation, motility, digestion, absorption, and electrolyte balance in gastrointestinal homeostasis. Disorganization of circadian rhythms leads to pathological processes and promotion of gastrointestinal disorders. One of the clock-controlled genes is *PERIOD* (*PER1*, *PER2* and *PER3*), which functions as a repressor of *CLOCK-BMAL1*-mediated transcription. *CLOCK-BMAL1* heterodimer enhances the expression of a reverse transcript of the erythroblastosis gene *NR1D1*, which in turn represses the positive clock element *Bmal1* expression [33, 34]. Further, *PER1* is important for the maintenance of circadian rhythms in cells. We observed upregulated *NR1D1* and *PER1,* and found upregulated levels of Basic helix-loop-helix family member e41 (*BHLHE41*) in duodenum samples from the FMT group at week 2. *BHLHE41* is indirectly regulated by the *CLOCK-BMAL1* heterodimer and functions as a transcriptional repressor and regulator of the circadian clock [35].

Importantly, *MMP-1* genes were upregulated in patients receiving FMT compared with placebo at weeks 2 and 16. *MMP-1* breaks down the interstitial collagens types I, II and III, and is associated with fibrosis in SSc [36]. It has been shown that fibrotic skin biopsies from SSc patients have a reduced level of *MMP-1* expression [37]. Upregulated mRNA expression levels of *MMP-1* genes in patients receiving FMT at weeks 2 and 16 may indicate *MMP-1* as a marker of altered cellular function. The changes observed at week 16 could be interpreted as an immunological effect in the duodenum after the initial stress response observed at week 2 in the FMT group. Interestingly, the genus *Bacteroides*, the family Lachnospiraceae, the genus *Agathobacter* and the genus *Phascolarctobactererium* were positively correlated with collagenase MMP1. Moreover, we observed strong positive correlation between *ITM2*, *PER1* and *SLC7A5* and the genus *Bacteroides* and the family Veillonellaceae, and between *KLF2* and the family Veillonellaceae at week 2. However, due to the low sample number, none of the changes in bacterial abundance were significantly correlated with the top up- and downregulated genes.

The present study is the first of its kind investigating the effect of FMT on duodenal samples from SSc patients, and even more importantly investigating the underlying mechanisms for GIT involvement in SSc. Notably, this is a descriptive, exploratory study with a small sample size; with the present calculations, type I and II error assessments and statistical analysis had limited applicability. Another limitation is the site of biopsy being in the duodenum, whereas the majority of GIT symptoms are reported to occur in the large intestines. As stated in the previous pilot trial, the endoscopic FMT administration is not ideal due to the associated risks; however, alternative options are not available to date. A concern regarding the safety of FMT was addressed after two immunocompromised patients contracted infections after FMT, with donor faeces containing drug-resistant *E. coli* [38, 39]. However, ACHIM is cultivated in the lab and is free of all human genes, viruses and antibiotic-resistance genes [39].

The findings of this study cannot be generalized to other SSc cohorts, as all of the ReSScue patients and a majority (59%) of the SSc controls were Caucasians with lcSSc. We performed bulk RNA-sequencing, in which gene expression analyses were across the whole cellular compartments but not within the individual cell types. Despite these limitations, this study provides a good indication of how FMT may exert its effect in SSc and of the analyses that should be conducted in a larger study.

In conclusion, we present the first explorative dataset, indicating effects of FMT by ACHIM on duodenal transcriptome profiling and cellular composition in SSc. We found that FMT-treatment with ACHIM might have an effect on duodenal inflammation, fibrosis, and lymphangiogenesis, and might correlate with improved patient-reported lower GIT symptoms, as reported in the previous ReSScue pilot trial [15]. We also identified for the first time changing expressions of several genes in duodenal samples of SSc patients receiving FMT.

## Supplementary Material

kead014_Supplementary_Data

## Data Availability

The data underlying this article will be shared on reasonable request to the corresponding author.
