# Supplementary material for: Effects of faecal microbiota transplantation on the small intestinal mucosa in systemic sclerosis
Source: Rheumatology (Oxford). 2023 Jan 23;62(8):2918–29. doi: 10.1093/rheumatology/kead014 (PMC10393441; doi:10.1093/rheumatology/kead014)
Supplement: kead014_Supplementary_Data [file kead014_supplementary_data.docx]

**SUPPLEMENTARY MATERIAL**

**Method:**

*Immunohistochemistry*

The duodenum biopsies from the ReSScue pilot patients and SSc control patients were collected at the Department of Gastroenterology at Oslo University Hospital. Duodenal biopsies from the SSc control patients were collected during duodenoscopy on clinical indication, while duodenal biopsies from the ReSScue pilot patients were collected during the ReSScue pilot study before FMT (week 0), and after FMT at week 2 and week 16. The biopsies were embedded in paraffin blocks and sectioned at 3 µm. Information of used antibodies are in Supplementary Table 1. Immunohistochemically (IHC) staining of Podoplanin (gp38, lymphatic vessel endothelial cells, and myofibroblasts), CD38 (activated T cells and plasma cells), α-Smooth muscle actin (αSMA, smooth muscle cells and myofibroblasts), CD64 (macrophages) was performed by an automated BOND MAX system (Leica) using the manufacturer`s Bond-Polymer-Refine-Detection kit containing peroxidase block, wash buffer, polymers-HRP, chromogen-substrate-DAB and haematoxylin for counterstaining (all from Leica). After deparaffinization, blocking of peroxidase and antigen-retrieval using citrate-buffer, the primary antibodies were used. Staining was visualized by the Bond Detection-Kit and covered by a mounting medium (Dako). Sirius Red was used to visualize collagen. Double staining of CD3 (T-cell receptors) and CD8 (T-cell co-receptors), and VEGFR3 (lymphatic endothelial cells) staining were performed on an automated Ventana Discovery ultra-system. For details, see Supplementary Table 1.

The stained slides were scanned with Axio Scan.Z1 (Zeiss) or Pannoramic MIDI II (3DHISTECH) and exported as .tiff files. Further, the exported files were analysed using ImageJ-win64 (Fiji) with a threshold adjusted for each staining. For αSMA, the positively stained tunica muscularis was adjusted manually in ImageJ-win64 before data processing. Staining ratios were calculated in Excel, and converted into fold change to facilitate the interpretation. The data was then visualized in GraphPad Prism 8 and pictures of the stained samples were taken with the Olympus DP80 Microscope in magnification 20x, 40x, and 100x. To simplify the interpretation of the staining, the mean fold change was calculated setting the baseline staining to one.

*RNA isolation*

Duodenal biopsies frozen on RNA-later from the ReSScue patients at week 0, 2 and 16 were sent to Genewiz Germany for Standard RNA-sequence analysis. RNA was isolated with Qiagen RNeasy Plus Universal Mini Kit and followed the manufacturer's instructions.

*Gene expression analysis*

P-values were calculated with the *Wald test*. Differential gene expression was considered significant for p-value ≤ 0.05. All genes showing p-value ≤ 0.05 for FMT week 0 and placebo week 0 were excluded from the comparative analyses at week 2 and 16. Gene expression in FMT group was considered downregulated with log2 fold change ≤ -0.5 and upregulated with log2 fold change ≥ 0.5 as compared normalized mean count between week 0 and 2 or 0 and 16, or between week 2 and 16. Next, the significant deregulated genes in the comparison between FMT and placebo groups between week 0 and 2 or 0 and 16, or between week 2 and 16, were used as input to the comprehensive gene set enrichment web server EnrichR (<https://maayanlab.cloud/Enrichr/>).

*Correlations with gut microbiota composition*

We assessed whether there were major differences in the gut microbiome composition at baseline between patients on different diets, which we could not identify. We performed correlation analysis with the relative abundance of one bacteria family (f. Lachnospiraceae) and four genera (g. Bacterioides, g. Dialister, g. Agathobacter and g. Phascolarctobacterium) with top upregulated genes at week 2. We aimed to correlate the changes in the bacteria and the expression of top upregulated genes at one time point (week 2), where we saw the positive effect of FMT. Similar correlations were made for the top deregulated proteins (data from IHC) and the same four bacteria genera at the same time point (week 2).

**Supplementary Table S1. IHC staining protocol and rational for application**

| **Staining** | **Host** | **Dilution** | **Company** | **System** | **Rational for marker** |
| --- | --- | --- | --- | --- | --- |
| gp38 | rat | 1:2000 | Thermo Fisher | Leica Bond-Max | Stains podoplanin, which is expressed on myofibroblasts ^1, 2^ and lymphatic endothelial cells ^3^ |
| CD38 | rabbit | 1:2000 | abcam | Leica Bond-Max | Activated T cells and plasma cells ^4, 5^ |
| αSMA | mouse | 1:1000 | Sigma | Leica Bond-Max & manually | Stains smooth muscle cells and myofibroblasts ^6^ |
| CD64 | mouse | 1:500 | abcam | Leica Bond-Max & manually | Stains macrophages ^7^ |
| VEGFR3 | goat | 1:200 | R&D | Discovery ULTRA | Stains lymphatic endothelial cells ^8^ |
| CD3/8 | rabbit | According to company instructions | Roche | Discovery ULTRA | Stains T-cell receptors/co-receptor for T-cell receptors ^9, 10^ |
| Sirius Red |  |  |  |  | Stains collagen ^11, 12^ |

**Supplementary Table S2. Bacteria genera associated with GI symptoms in different diseases**

| Bacteria genera | Prior studies |
| --- | --- |
| *g. phascolarctobactereriae* | Decreased in IBD patients ^13^ |
| *g. Bacteroides* | Protects from intestinal mucosal inflammation and invasion of pathogenic species in Crohn’s disease patients ^14^, and is decreased in SSc patients ^15^. |
| *g. Dialister* | Positively correlates between bacteria and fibrosis severity in non-obese NAFLD patients, making the bacteria potentially pro-fibrotic ^16^ |
| *g. Agathobacter* | Depleted in non-obese NAFLD patients ^16^, and might have anti-fibrotic effects. |

**Supplementary Figure S1. HE staining of duodenal tissue from ReSScue pilot trial patients**

a) HE staining of duodenal biopsy from ReSScue trial patient receiving placebo and b) ReSScue trial patient receiving FMT.


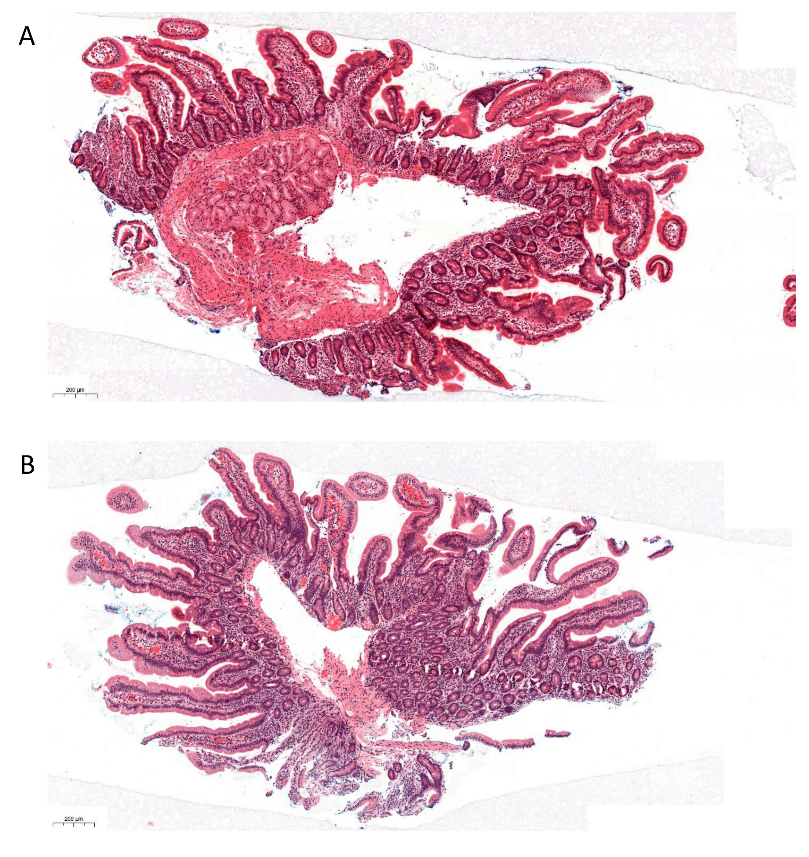


**Supplementary Table S3. Histological assessment of HE stained duodenal tissue from ReSScue pilot trial patients**

|  | Presence of duodenal structures | | | |
| --- | --- | --- | --- | --- |
| Week | Intestinal villi | Crypts | Mucosa | Submucosa |
| 0, n/N (%) | 8/8 (100) | 8/8 (100) | 5/8 (62·5)^*^ | 3/8 (37·5)^†^ |
| 2, n/N (%) | 9/9 (100) | 9/9 (100) | 9/9 (100) | 2/9 (22.2)^†^ |
| 16 n/N (%) | 6/6 (100) | 6/6 (100) | 5/6 (83·3)^*^ | 4/6 (66·7)^†^ |

*: Not fully represented, †: Not fully represented

**Supplementary Figure S2.** **Immunohistochemical staining of duodenal samples of ReSScue patients (FMT n=5, Placebo n=4)**

(a-e) Additional pictures of IHC staining for Podoplanin, CD64, CD38, VEGFR3 and CD3/8 co-staining at week 0 and 2 in magnification x40 and x100.


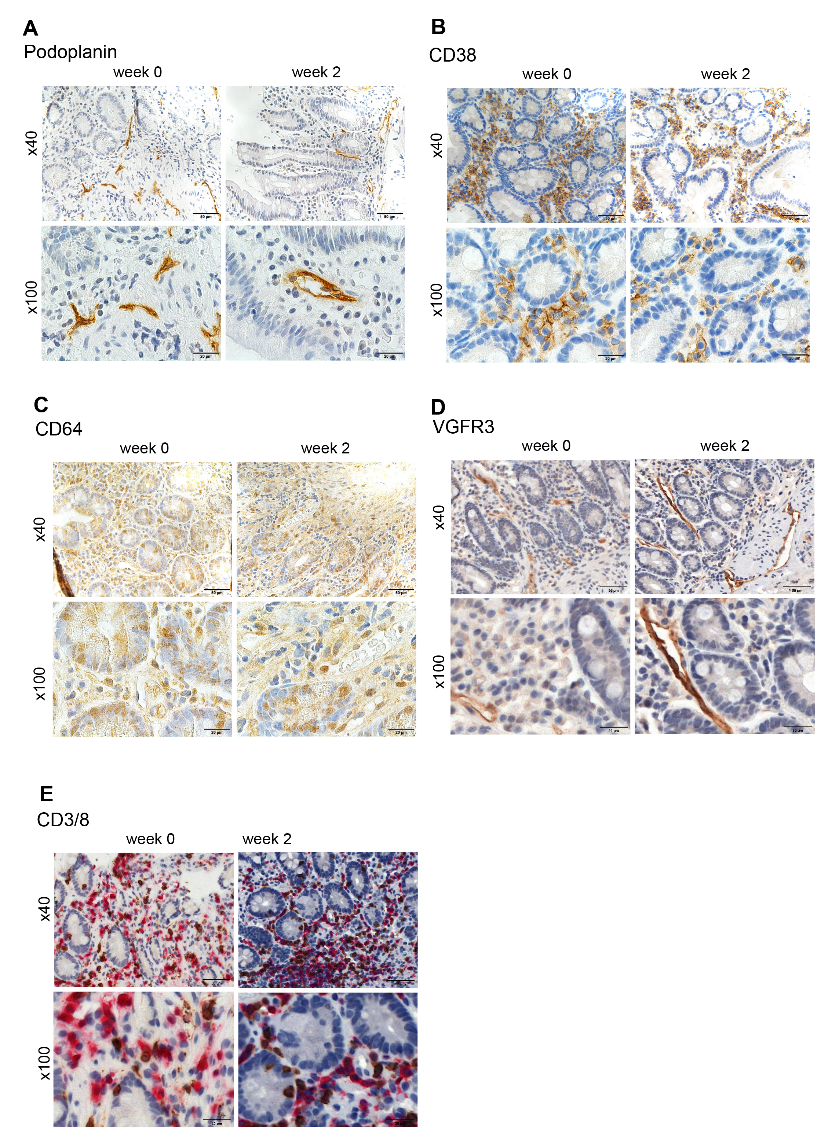


**Supplementary Figure S3. Clinical correlation of staining ratios of FMT and placebo treated patients.** Fold change of staining ratios of podoplanin (a) and CD64 (b) correlated to lower UCLA GIT Score at week 0, 2 and 16. Patients who received FMT (n=5) in blue and placebo (n=4) in black (*Pearson correlation*).


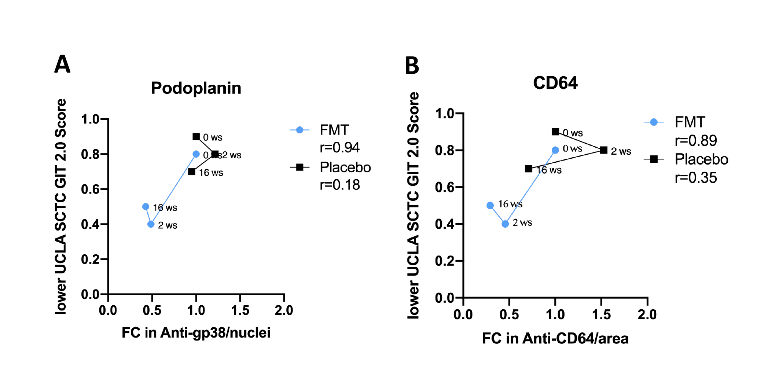


**Supplementary Figure S4. Correlation of staining ratios and relative abundance of bacteria in FMT and placebo treated patients.** Fold change of staining ratios of podoplanin (green) and CD64 (pink) correlated with relative abundance (%) of g. Bacterioides (A), g. Dialister (B), f. Lachnospiraceae (C), g. Agathobacter (D) and g. Phascolarctobacterium (E) at week 0, 2 and 16 in patients receiving Placebo and ACHIM.

**

**

**Supplementary Figure S5. Transcriptomic analysis of duodenum samples from SSc Placebo and FMT patients at week 2**

Bulk RNA sequencing was performed on duodenum biopsies from SSc Placebo (4) versus FMT (5) patients. a) The heat map and (b-d) pathway enrichment analysis of significantly downregulated genes in duodenum biopsies from FMT versus Placebo patients at week 2. Pathway enrichment analysis of differentially expressed genes (p≤0.05, log_2_ratio ≥-0.5) was performed with the Enrichr software.


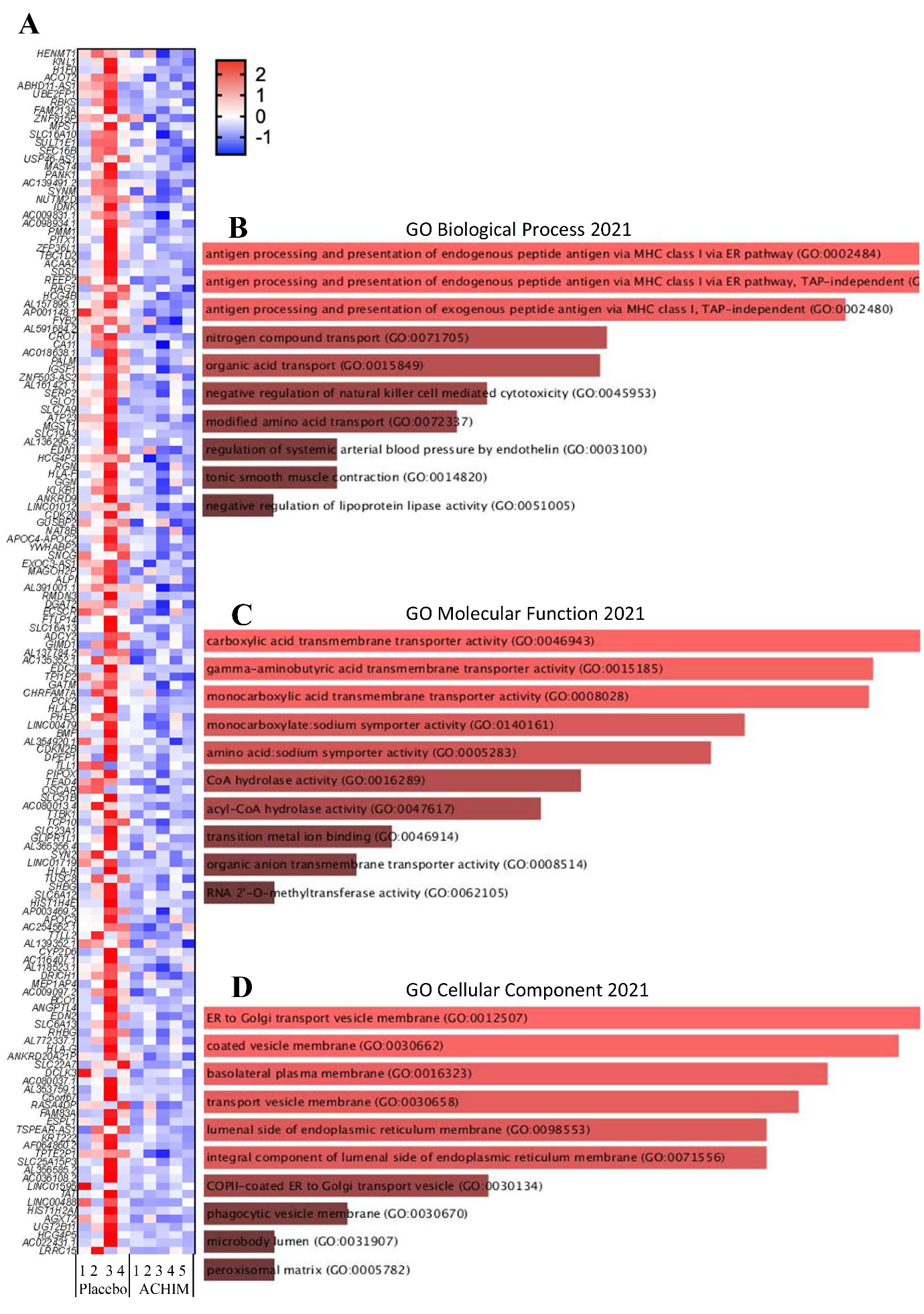


**Supplementary Figure S6. Transcriptomic analysis of duodenum samples from SSc Placebo and FMT patients at week 16**

Bulk RNA sequencing was performed on duodenum biopsies from SSc Placebo (4) versus FMT (5) patients. a) The heat map and (b-d) pathway enrichment analysis of significantly upregulated genes in duodenum biopsies from FMT versus Placebo patients at week 16. Pathway enrichment analysis of differentially expressed genes (p≤0.05, log_2_ratio ≥0.5) was performed with the Enrichr software.


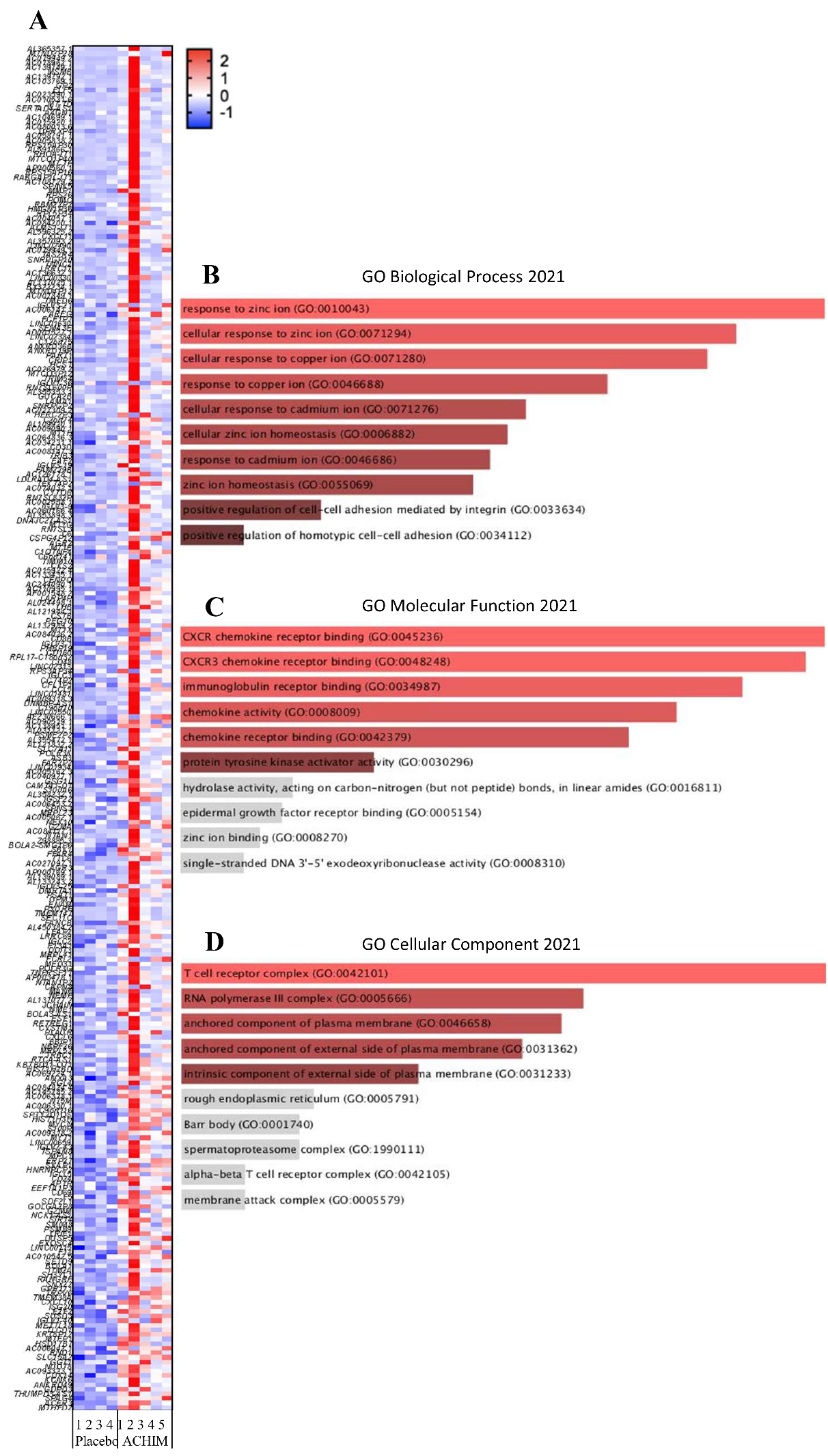


**Supplementary Figure S7. Transcriptomic analysis of duodenum samples from SSc Placebo and FMT patients at week 16**

Bulk RNA sequencing was performed on duodenum biopsies from SSc Placebo (4) versus FMT (5) patients. a) The heat map and (b-d) pathway enrichment analysis of significantly downregulated genes in duodenum biopsies from FMT versus Placebo patients at week 16. Pathway enrichment analysis of differentially expressed genes (p≤0·05, log_2_ratio ≥-0·5) was performed with the Enrichr software.


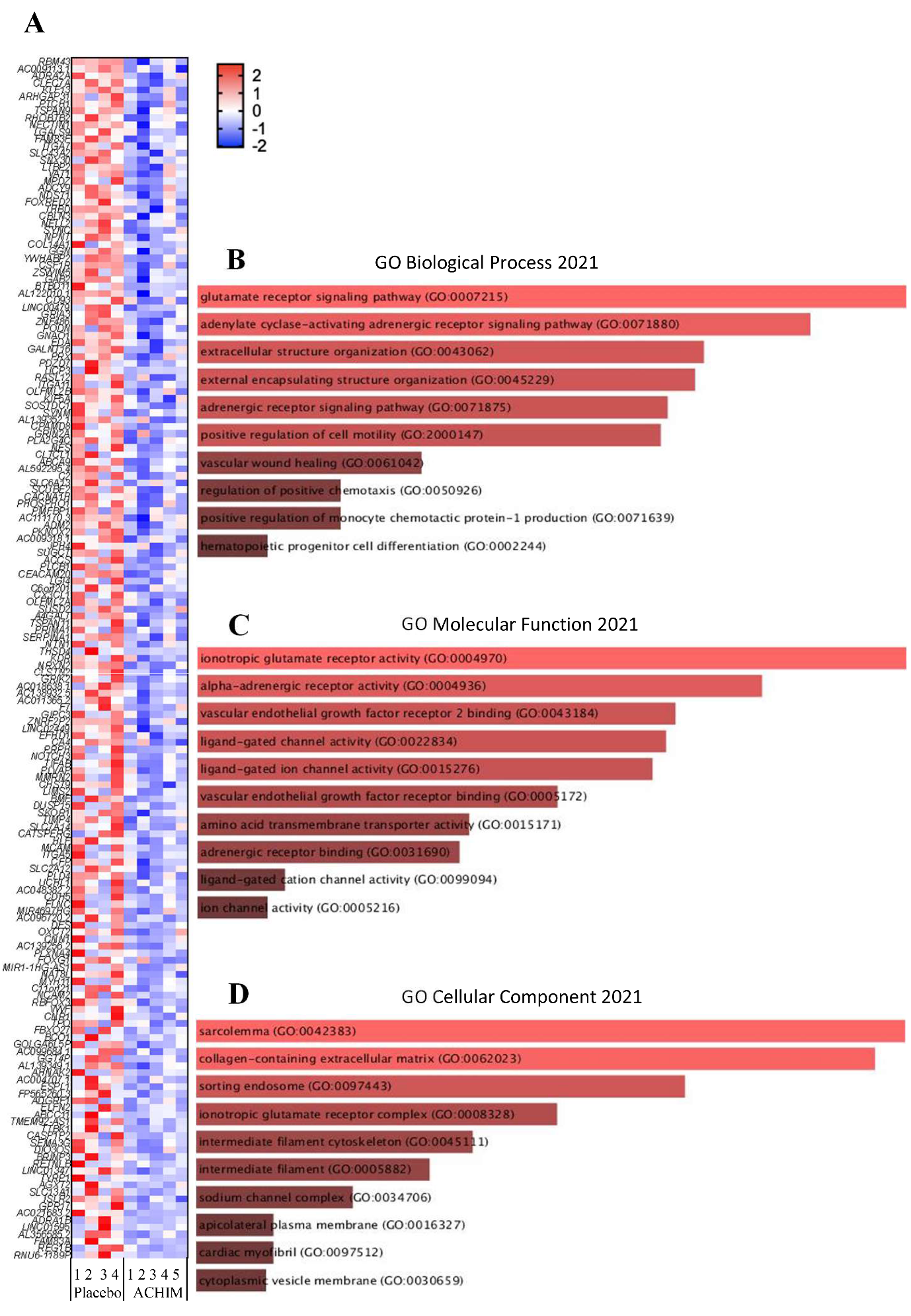


**Supplementary Figure S8. Transcriptomic comparative analysis of duodenum samples from SSc Placebo and FMT patients between week 2 and 16**

Analysis was performed with the comprehensive gene set enrichment web server EnrichR for *Pathways* (database: A) Reactome 2016, B) KEGG 2021 Human) of significantly downregulated genes in FMT samples from week 2 and 16.


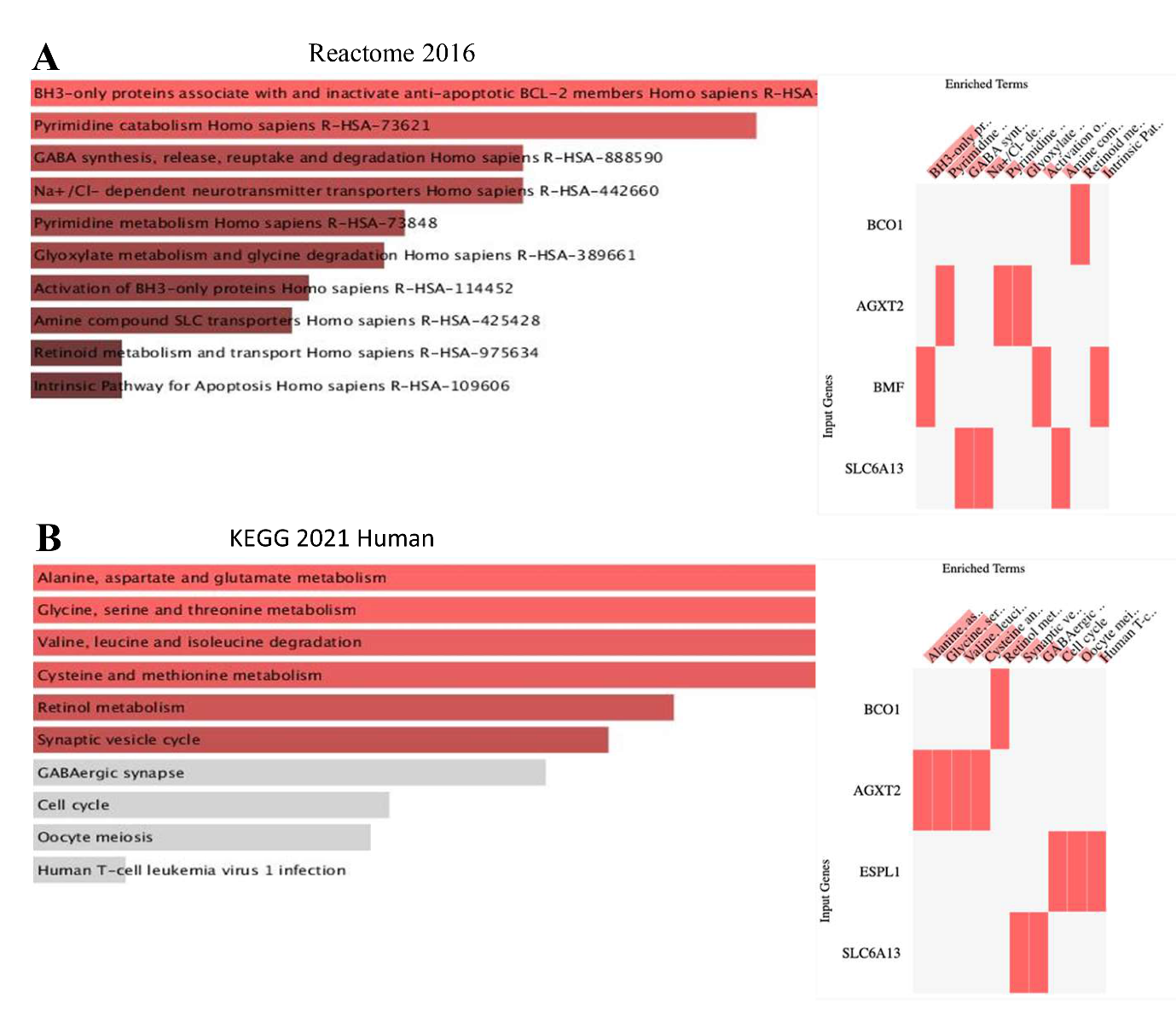


**Supplementary Figure S9. Correlations between gut microbiome composition and the changes in transcriptome profiling.** (A-E) Spearman correlation between the relative abundance (%) of bacteria (g. Bacterioides (A), g. Dialister (B), f. Lachnospiraceae (C), g. Agathobacter (D) and g. Phascolarctobacterium (E) and the changes in selected gene expression levels in duodenal samples from SSc patients treated with FMT or placebo at week 0, 2 and 16.

**

**

**References:**

1. Stellato M, Rudnik M, Renoux F, Pachera E, Sotlar K, Klingel K, et al. OP0049 Myocardial Fibrogenesis in Systemic Sclerosis: Involvement of A Novel Stromal Sub-Population. Annals of the rheumatic diseases. 2016;75(Suppl 2):73-.

2. Nazari B, Rice LM, Stifano G, Barron AM, Wang YM, Korndorf T, et al. Altered dermal fibroblasts in systemic sclerosis display podoplanin and CD90. The American journal of pathology. 2016;186(10):2650-64.

3. Manetti M, Milia AF, Guiducci S, Romano E, Matucci-Cerinic M, Ibba-Manneschi L. Progressive Loss of Lymphatic Vessels in Skin of Patients with Systemic Sclerosis. The Journal of Rheumatology. 2011;38(2):297-301.

4. Soto L, Ferrier A, Aravena O, Fonseca E, Berendsen J, Biere A, et al. Systemic Sclerosis Patients Present Alterations in the Expression of Molecules Involved in B-Cell Regulation. Frontiers in immunology. 2015;6:496-.

5. Piedra-Quintero ZL, Wilson Z, Nava P, Guerau-de-Arellano M. CD38: An Immunomodulatory Molecule in Inflammation and Autoimmunity. Frontiers in Immunology. 2020;11.

6. Rajkumar VS, Howell K, Csiszar K, Denton CP, Black CM, Abraham DJ. Arthritis Research & Therapy. 2005;7(5):R1113.

7. Stifano G, Christmann RB. Macrophage Involvement in Systemic Sclerosis: Do We Need More Evidence? Current Rheumatology Reports. 2016;18(1).

8. Didriksen H, Molberg Ø, Fretheim H, Gude E, Jordan S, Brunborg C, et al. Association of Lymphangiogenic Factors With Pulmonary Arterial Hypertension in Systemic Sclerosis. Arthritis & Rheumatology. 2021;73(7):1277-87.

9. Fuschiotti P. Current perspectives on the role of CD8+ T cells in systemic sclerosis. Immunol Lett. 2018;195:55-60.

10. O'Reilly S, Hugle T, Van Laar JM. T cells in systemic sclerosis: a reappraisal. Rheumatology. 2012;51(9):1540-9.

11. Lattouf R, Younes R, Lutomski D, Naaman N, Godeau G, Senni K, et al. Picrosirius Red Staining. Journal of Histochemistry & Cytochemistry. 2014;62(10):751-8.

12. Schniering J, Guo L, Brunner M, Schibli R, Ye S, Distler O, et al. Evaluation of 99mTc-rhAnnexin V-128 SPECT/CT as a diagnostic tool for early stages of interstitial lung disease associated with systemic sclerosis. Arthritis Research & Therapy. 2018;20(1).

13. Morgan XC, Tickle TL, Sokol H, Gevers D, Devaney KL, Ward DV, et al. Dysfunction of the intestinal microbiome in inflammatory bowel disease and treatment. Genome Biol. 2012;13(9):R79.

14. Schäffler H, Herlemann DPR, Alberts C, Kaschitzki A, Bodammer P, Bannert K, et al. Mucosa-attached bacterial community in Crohn's disease coheres with the clinical disease activity index. Environ Microbiol Rep. 2016;8(5):614-21.

15. Volkmann ER, Hoffmann-Vold A-M, Chang Y-L, Jacobs JP, Tillisch K, Mayer EA, et al. Systemic sclerosis is associated with specific alterations in gastrointestinal microbiota in two independent cohorts. BMJ Open Gastroenterology. 2017;4(1):e000134.

16. Lee G, You HJ, Bajaj JS, Joo SK, Yu J, Park S, et al. Distinct signatures of gut microbiome and metabolites associated with significant fibrosis in non-obese NAFLD. Nat Commun. 2020;11(1):4982.
